# Clinicopathological features and immune checkpoint expression patterns in dMMR early gastric cancer: a retrospective pilot study

**DOI:** 10.1186/s12885-026-16298-3

**Published:** 2026-06-15

**Authors:** Yusaku Yokotani, Naoyuki Sakamoto, Mikiko Matsuo, Yuki Nakahata, Haruka Okuyama, Rieko Mukai, Junji Nagano, Akihiro Obora, Yoshiki Murakami, Takao Kojima, Nobuaki Yagi

**Affiliations:** 1 Department of Gastroenterology, Asahi University Hospital, Gifu, Japan; 2 Department of Pathology, Asahi University Hospital, Gifu, Japan

**Keywords:** dMMR, Early gastric cancer, Immune checkpoint, PD-L1, LAG-3, TIM-3, Tumor microenvironment

## Abstract

**Background:**

Although mismatch repair–deficient (dMMR)/microsatellite and instability–high (MSI-H) status is recognized as a biomarker that predicts the efficiency of immune checkpoint inhibitors (ICIs), the efficacy of ICIs treatment is still insufficient. Accordingly, insights into the mechanisms of acquired resistance to ICIs in gastrointestinal cancers are necessary, and developing immunotherapeutic strategies to improve response rates is an urgent clinical priority.

We investigated the clinical characteristics of dMMR/MSI-H early gastric cancer and analyzed the expression patterns of immune checkpoint molecules within the tumor microenvironment.

**Method:**

A total of 20 cases of early gastric cancer, treated between November 2019 and July 2024, were included. Immunohistochemistry was performed to assess the expression of mismatch repair (MMR) protein, and patient clinical characteristics were evaluated. Additionally, among the eight cases identified as dMMR early gastric cancer, they were further examined for the expression of immune checkpoint molecules in the tumor microenvironment using immunohistochemical staining.

**Result:**

The median age of patients diagnosed with dMMR early gastric cancer was 73 years. In 20 patients, female sex and severe atrophy were associated with dMMR status in univariate analysis (OR 7.67, 95% CI 1.04–94.53). The expression pattern of immune checkpoint molecules showed heterogeneity.

**Conclusion:**

These findings highlight the heterogeneity of immune checkpoint expression patterns and provide preliminary, hypothesis-generating insights into variability in response to PD-1/PD-L1 blockade.

## Introduction

Importantly, mismatch repair–deficient (dMMR)/microsatellite instability–high (MSI-H) status has emerged as a predictive biomarker for immune checkpoint inhibitor (ICI) therapy across solid tumors, including gastric cancer (GC). Additionally, the identification of molecular subtypes, particularly MSI-H or dMMR tumors, has provided new insights into the biology of several types of cancer. Current guidelines, therefore, recommend routine MSI/MMR testing to individualize treatment and highlight the potential role of immunotherapy in this setting [1].

dMMR/MSI-H GC is a distinct subgroup with a favorable prognosis but reduced sensitivity to cytotoxic chemotherapy [2]. Although perioperative chemotherapy improves survival in resectable GC overall, subgroup analyses of the MAGIC trial showed no benefit, and possibly worse outcomes, in dMMR/MSI-H patients [3]. A recent meta-analysis likewise found no significant improvement in overall or disease-free survival with perioperative chemotherapy [8]. Therefore, a deeper characterization of dMMR/MSI-H GC is needed to identify subgroups in which chemotherapy may be unnecessary, and immunotherapy may serve as the optimal treatment strategy.

A thorough understanding of the underlying immunological mechanisms is essential when considering cancers that exhibit the characteristics of dMMR/MSI-H GC. Recent studies have suggested that, in addition to PD-1/PD-L1, other immune checkpoint molecules, such as lymphocyte-activation gene 3 (LAG-3) and T-cell immunoglobulin and mucin-domain containing-3 (TIM-3), play critical roles in shaping the tumor immune microenvironment and may contribute to ICI resistance [4–6].

The objective response rate (ORR) of ICI monotherapy in patients with dMMR/MSI-H colorectal cancer has been reported to be as high as 45.8% [7]. Although dMMR/MSI-H gastric cancer is considered an immunologically active subtype, clinical responses to PD-1/PD-L1 blockade remain heterogeneous. To maximize the efficacy of immunotherapy and to establish individualized combination treatment strategies, comprehensive immunological analyses of the tumor microenvironment in dMMR/MSI-H solid tumors are considered essential.

dMMR/MSI-H EGC has a relatively higher incidence than advanced gastric cancer [8], and early-stage tumors may reflect intrinsic immune regulatory programs before therapy-induced or evolutionary immune remodeling, providing insight into primary resistance mechanisms. Therefore, it was considered an appropriate model for investigating the immunological characteristics of the tumor microenvironment in dMMR/MSI-H EGC. Given the limited number of available cases, this study was designed as an exploratory analysis to generate hypotheses regarding immune checkpoint architecture in early-stage dMMR/MSI-H gastric cancer.

## Materials and methods

### Patient characteristics and sample collection

A retrospective single-center study was conducted to characterize clinicopathological features and immune checkpoint expression in dMMR early gastric cancer. A total of 20 patients with histologically confirmed early gastric cancer who underwent radical gastrectomy or endoscopic submucosal dissection (ESD) at Asahi University Hospital between November 2019 and July 2024 were included.

All cases underwent immunohistochemical (IHC) staining for MMR proteins. Inclusion criteria were histologically confirmed early gastric cancer with available tissue specimens suitable for immunohistochemical evaluation.

Clinical, pathological, and endoscopic characteristics were obtained from medical records. The study was conducted in accordance with the Declaration of Helsinki and approved by the institutional ethics committee.

### Immunohistochemistry

**Fig. 1:**
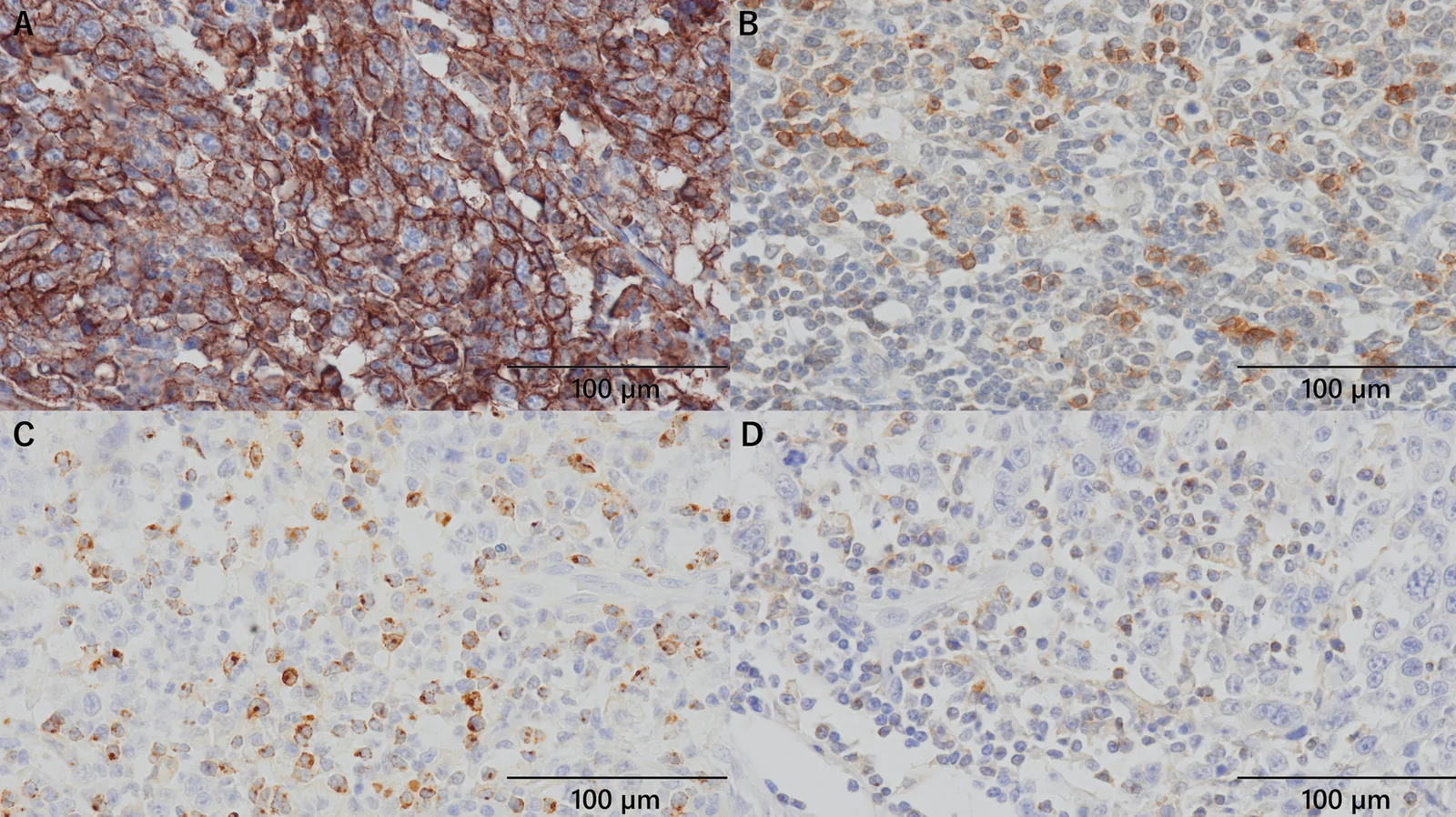
Representative images of PD-L1 expression, PD-1 infiltration, LAG-3,and TIM-3 staining. Immunohistochemistry showed positive staining of PD-L1 (**A**), PD-1 (**B**), LAG-3 (**C**), TIM-3 (**D**). All images were obtained at ×200 magnification

20 samples underwent IHC staining for DNA mismatch repair (MMR) proteins. MMR protein expression was analyzed by IHC using antibodies against MLH1, PMS2, MSH2, and MSH6. Accordingly, 8 cases in EGC were diagnosed as dMMR early gastric cancer. The IHC staining for immune checkpoint molecules (PD-L1, PD-1, TIM-3, LAG-3) was conducted on these 8 cases. The representative images of these IHC staining were provided in Fig. 1.

## Immunohistochemical analysis of MMR and PD-L1, PD-1, LAG-3, and TIM-3

Cases identified as not only showing concurrent loss of both MLH1 and PMS2 but also being older patients were regarded as sporadic dMMR cases. Expression of programmed death-ligand 1 (PD-L1) was assessed using the Combined Positive Score (CPS), which represents the percentage of PD-L1-positive tumor and tumor-infiltrating immune cells among total viable cells. PD-L1 expression was assessed by immunohistochemistry using the PD-L1 IHC 22C3 pharmDx assay with an anti–PD-L1 mouse monoclonal antibody (clone 22C3, Agilent Technologies/Dako, Santa Clara, CA, USA) on the Dako Autostainer Link 48 platform. PD-L1 expression was evaluated using the combined positive score (CPS), defined as the number of PD-L1–positive tumor cells, lymphocytes, and macrophages divided by the total number of viable tumor cells, multiplied by 100. A CPS ≥ 1 was considered positive, and CPS ≥ 10 was defined as high expression.

Immunohistochemical expression of PD-1, LAG-3, and TIM-3 was evaluated in tumor-infiltrating immune cells within the tumor microenvironment. For PD-1, intratumoral infiltration was assessed and categorized as positive, focal/weak, or negative based on the extent and intensity of membranous staining in tumor-infiltrating lymphocytes. For LAG-3 and TIM-3, expression was evaluated based on the presence and distribution of staining-positive immune cells. In particular, “hot spots” were defined as focal areas with a visibly higher density of positive immune cells compared to the surrounding regions (Fig. 1).

Based on these observations, expression patterns were categorized descriptively as follows:

- Positive: clear and dense accumulation of staining-positive immune cells, including identifiable hot spots.
- Rare positive: only a small number of scattered positive immune cells without distinct clustering.
- Scattered rare positive: very sparse and diffusely distributed positive cells without focal accumulation.

Because standardized cutoff values for these immune checkpoint molecules have not been established in early gastric cancer, these categorizations were defined descriptively for the purpose of this exploratory analysis.

### Endoscopic and imaging assessment

Conventional white-light endoscopy was used in all cases, and when available, image-enhanced modalities such as narrow-band imaging or chromoendoscopy were also reviewed.

Macroscopic morphology of the lesions was classified using the Paris endoscopic classification and the Japanese-Borrmann classification.

Lesion location within the stomach was categorized into upper (U), middle (M), or lower (L) third according to the Japanese classification system. Endoscopic ultrasonography (EUS) was performed in selected cases to estimate the depth of invasion and to discriminate mucosal from submucosal lesions. When EUS was not available, the depth of invasion was estimated based on endoscopic appearance, including the presence of ulceration, fold convergence, and non-lifting signs. For patients undergoing ESD, the indication criteria followed the Japanese Gastric Cancer Association guidelines, including absolute and expanded indications. En bloc resection, R0 resection, and curative resection rates were recorded.

### Statistical analysis

Descriptive analyses were performed to summarize clinicopathological characteristics. All statistical analyses were performed using R software (R Foundation for Statistical Computing, Vienna, Austria) in the RStudio environment (Posit Software, Boston, MA, USA). Continuous variables were summarized as median (range), and categorical variables were summarized as counts and percentages. Given the limited sample size and presence of sparse data, associations between clinicopathological variables and dMMR status were evaluated using Firth penalized logistic regression to obtain bias-reduced estimates.

## Results

### Clinicopathological characteristics of dMMR/MSI-H early gastric cancer

**Fig. 2:**
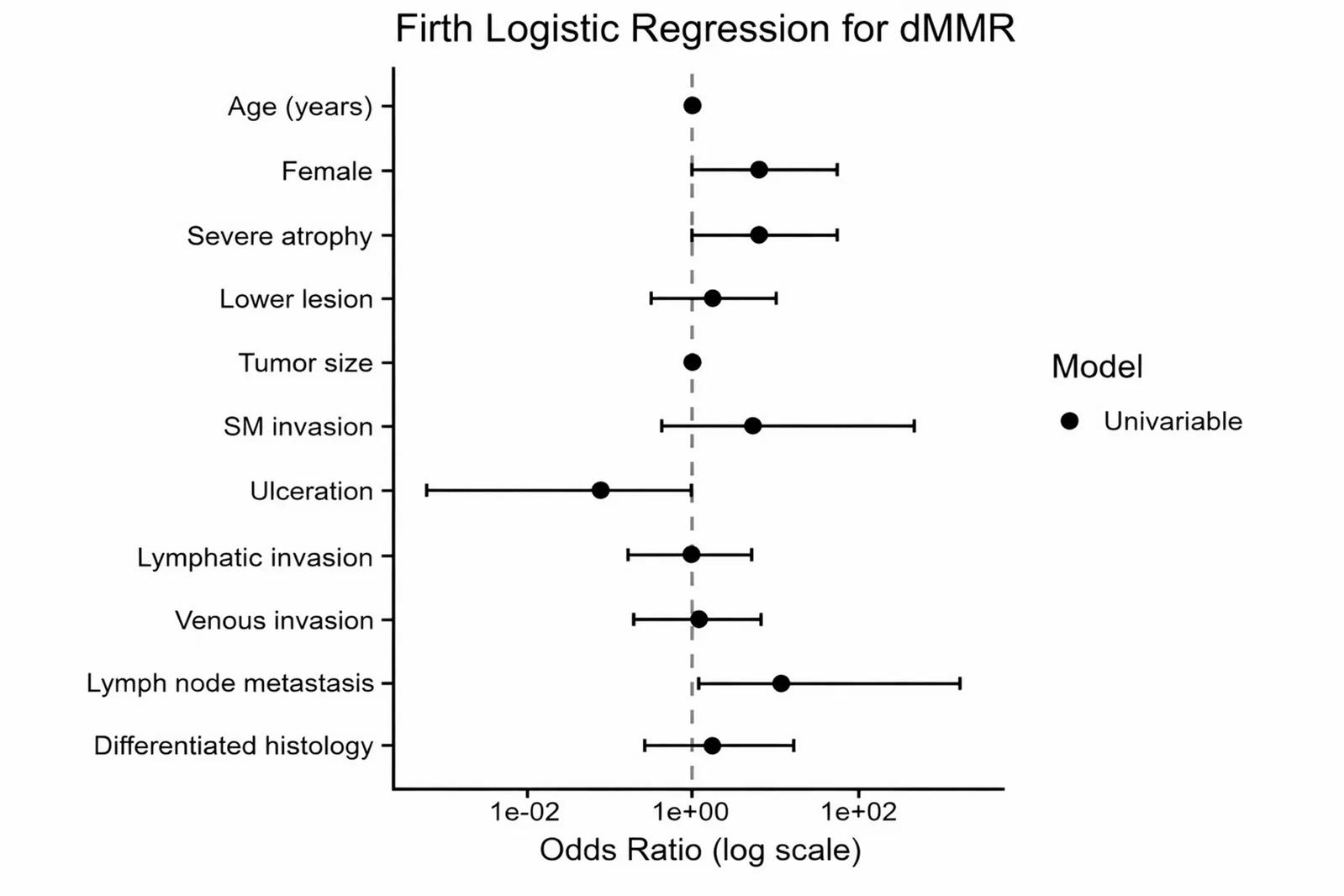
Forest plot of theFirth logistic regression analysis for dMMR. Odds ratios (ORs) and 95% confidence intervals (CIs) for each clinicopathological variable are shown on a logarithmic scale. Black circles represent results from univariable Firth logistic regression analyses, and red diamonds indicate multivariable-adjusted estimates. Firth penalized likelihood was applied to reduce bias associated with small sample size and potential separation. The dashed vertical line indicates the null value (OR = 1)

A total of 20 patients were included in the analysis. Baseline clinicopathological characteristics are shown in Table 1 without any hypothesis testing, due to the small sample size. Firth penalized logistic regression was used to evaluate the association between clinical variables and dMMR status, accounting for the small sample size and several variables, such as ulceration and lymph node metastasis, that demonstrated quasi-complete separation, resulting in unstable estimates. In univariate analysis, female sex and severe atrophy were significantly associated with dMMR in Table 2 (odds ratio [OR] 7.67, 95% confidence interval [CI] 1.04–94.53; *p* = 0.045). This analysis for all the factors was interpreted in the Forest plot (Fig. 2). Female sex and severe background mucosal atrophy demonstrated identical estimates in univariable Firth regression analysis, suggesting substantial collinearity and overlapping distributions within the study cohort.

**Table 1:** Baseline characteristics

		dMMR status	
Variable	Overall *N* = 20	pMMR *N* = 12	dMMR *N* = 8
Age (years)	74.0 (68.0, 79.5)	74.0 (67.5, 79.5)	73.0 (69.0, 82.0)
Sex			
male	15 (75%)	11 (92%)	4 (50%)
female	5 (25%)	1 (8.3%)	4 (50%)
Tumor size (mm)	25.0 (22.5, 33.0)	25.0 (16.5, 30.0)	28.5 (25.0, 37.0)
Depth			
M	3 (15%)	3 (25%)	0 (0%)
SM	17 (85%)	9 (75%)	8 (100%)
Lesion			
Upper, Middle	7 (35%)	5 (42%)	2 (25%)
Lower	13 (65%)	7 (58%)	6 (75%)
Ulceration			
No	15 (75%)	7 (58%)	8 (100%)
Yes	5 (25%)	5 (42%)	0 (0%)
Lymph node metastasis			
No	17 (85%)	12 (100%)	5 (63%)
Yes	3 (15%)	0 (0%)	3 (38%)
Lymphatic invasion			
No	10 (50%)	6 (50%)	4 (50%)
Yes	10 (50%)	6 (50%)	4 (50%)
Venous invasion			
No	13 (65%)	8 (67%)	5 (63%)
Yes	7 (35%)	4 (33%)	3 (38%)
Differentiated histology			
No	4 (20%)	3 (25%)	1 (13%)
Yes	16 (80%)	9 (75%)	7 (88%)

Data are presented as median (interquartile range) or number (percentage), as appropriate. Baseline characteristics are described descriptively without formal hypothesis testing due to the small sample size

**Table 2:** Univariable firth logistic regression analysis for dMMR

Variable	OR	95% CI	*p*
Age	1.02	0.9–1.16	0.725
Female	7.67	1.04–94.53	0.045
Severe atrophy	7.67	1.04–94.53	0.045
Lower lesion	1.91	0.32–13.77	0.483
Tumor size	1.00	0.97–1.03	0.953
SM invasion	6.26	0.5–889.85	0.173
Ulceration	0.08	0–0.91	0.040*
Lymphatic invasion	1.00	0.18–5.63	1.000
Venous invasion	1.20	0.2–7.17	0.838
Lymphnode metastasis	15.91	1.24–2285.06	0.032*
Differentiated histology	1.84	0.24–22.13	0.567

Variables showing complete or quasi-complete separation (e.g., ulceration and lymh node metastasis) may produce inflated odds ratios and wide confidence intervals despite penalized estimation; therefore, these results should be interpreted cautiously and considered exploratory

*Interpret with caution due to separation

### Immune checkpoint expression in the tumor microenvironment

IHC evaluation of immune checkpoint molecules revealed that PD-1–positive tumor-infiltrating lymphocytes and PD-L1–positive tumor or immune cells were present in a subset of dMMR early gastric cancers. Using CPS-based scoring, several cases exhibited a PD-L1 CPS > 10, indicating high PD-L1 expression (Table 3). These findings suggest that immune evasion through the PD-1/PD-L1 axis may be established even at an early stage of tumor development in dMMR lesions.

**Table 3:** Descriptive summary of immune checkpoint expression patterns in dMMR early gastric cancer

Case	PD-L1 CPS category	Intratumoral PD-1+ infiltration	Peritumoral lymphoid aggregates (%)	LAG-3 hot spot	TIM-3 hot spot
1	High (≥10)	Positive	10	Positive	Positive
2	High (≥10)	Positive	10–20	Positive	Positive
3	High (≥10)	Focal/weak	5–10	Positive	Rare positive
4	Intermediate (1–<10)	Negative	0–1	Positive	Rare positive
5	Intermediate (1–<10)	Negative–focal	0–10	Rare positive	Rare positive
6	Intermediate (1–<10)	Negative	0–2	Rare positive	Scattered rare positive
7	Intermediate (1–<10)	Negative	0–1	Rare positive	Scattered rare positive
8	Low (<1)	Negative	0–1	Rare positive	Rare positive

Eight cases were analyzed for PD-L1 CPS, intratumoral PD-1 + cell infiltration, PD-1 expression in peritumoral lymphoid aggregates, LAG-3, and TIM-3 expression. Given the exploratory nature and small sample size, the findings are presented descriptively for each case

In addition to PD-1 and PD-L1, expression of other inhibitory checkpoint molecules, including TIM-3, and LAG-3 was detected on infiltrating lymphocytes and macrophages within the tumor nests and at the invasive front in varying degrees (Table 3). The coexpression of multiple immune checkpoints in the tumor microenvironment supports the concept of a complex diversity in immune-checkpoint molecules.

## Discussion

This study demonstrated that dMMR/MSI-H early gastric cancer exhibits substantial heterogeneity in immune checkpoint expression profiles within the tumor microenvironment. Although dMMR/MSI-H status is generally considered an immunologically active phenotype, our findings suggest that immune regulatory architectures vary considerably even within this molecular subgroup, indicating that MSI-H status alone may not fully define immune responsiveness.

Importantly, while previous studies have predominantly focused on advanced or metastatic disease, the present analysis highlights that diverse immune checkpoint co-expression patterns are already established at an early stage of tumor development. This observation raises the possibility that intrinsic immune regulatory programs may contribute to primary differences in therapeutic sensitivity, independent of treatment-induced immune remodeling.

Careful recognition of GC’s clinical characteristics is crucial to avoid missing cases of dMMR/MSI-H tumors. Previous studies have described characteristic clinicopathological features of dMMR/MSI-H early gastric cancer, including a female predominance, distinct morphological features, and frequent occurrence in severely atrophic mucosa [8–10].

dMMR/MSI-H gastrointestinal cancers constitute a molecular subtype that tends to respond favorably to immune checkpoint blockade. Nevertheless, therapeutic outcomes remain heterogeneous, and resistance can arise through multiple mechanisms.

The post-hoc analysis of the KEYNOTE-059/061/062 studies reported a relatively high objective response rate in patients with MSI-H gastric or gastroesophageal junction cancer treated with pembrolizumab, a subset of patients still failed to achieve sufficient clinical benefit [11].

Continued investigation focusing on this tumor subset is therefore warranted, with the dual aims of clarifying the biological basis of immune resistance and advancing the development of rational combination immunotherapies incorporating ICIs. For example, understanding T cell exhaustion is important for discussing the immune checkpoint molecules.

T cell exhaustion represents a key mechanism limiting durable anti-tumor immunity and is characterized by sustained expression of multiple inhibitory receptors, including PD-1, LAG-3, and TIM-3 [12]. Although PD-1/PD-L1 blockade has significantly improved cancer treatment outcomes, responses remain heterogeneous, suggesting that additional inhibitory pathways contribute to persistent immune dysfunction [13, 14].

Consistent with previous reports, LAG-3 and TIM-3 are frequently co-expressed with PD-1 on exhausted tumor-infiltrating lymphocytes and may reflect more advanced states of immune regulation [15–17]. In particular, upregulation of alternative checkpoints such as TIM-3 has been implicated in adaptive resistance following PD-1 blockade [18]. These findings support a model in which T cell exhaustion is maintained by a network of overlapping inhibitory pathways rather than a single dominant checkpoint [19].

In the present study, heterogeneous co-expression patterns of PD-1, PD-L1, LAG-3, and TIM-3 were observed in dMMR early gastric cancer, suggesting that diverse immune regulatory states may already be established at an early stage of tumor development. This heterogeneity may partly explain variability in immune responsiveness within the dMMR subgroup and provides a rationale for exploring combination immunotherapeutic strategies. However, given the exploratory nature of this study, these interpretations should be considered hypothesis-generating.

In contrast to previous reports, no factors except female, associated with dMMR status were identified in this study, which may be partly due to the small sample size. On the other hand, findings indicate that the expression pattern of immune checkpoint molecules is heterogeneous, and this may be associated with the acquisition of resistance to ICI treatment. From a clinical perspective, these findings suggest that evaluation of checkpoint co-expression patterns, rather than reliance solely on MSI-H status, may help refine immunotherapeutic strategies. However, given the exploratory design and limited sample size, these results should be interpreted as hypothesis-generating and require validation in larger cohorts.

Despite these potential implications, several limitations of this study should be acknowledged. This study has several important limitations. First, the sample size was small, with only 20 patients included and 8 cases classified as dMMR, which limits the statistical power and reliability of the analyses with the possibility of Type I error. Therefore, the results of the regression analyses should be interpreted with caution and are considered exploratory and hypothesis-generating rather than confirmatory. Second, this was a single-center retrospective study, and cases were not consecutively enrolled but were selected based on the availability of tissue specimens suitable for immunohistochemical evaluation. This may have introduced selection bias. In addition, the study population consisted of relatively elderly patients (median age: 74 years), which may have further influenced the observed clinicopathological characteristics, including the relatively high prevalence of dMMR compared to previous reports. Third, due to the limited sample size, we were unable to perform multivariable analyses without the risk of overfitting. Accordingly, the results of the regression analyses should not be interpreted as identifying independent predictors. Fourth, this study focused on descriptive immunohistochemical evaluation of immune checkpoint expression. Functional validation, survival analysis, treatment response data, and correlation with clinical outcomes were not performed. Therefore, the biological and clinical significance of the observed heterogeneity in immune checkpoint expression remains to be determined.

Finally, given the exploratory nature of this study, further validation in larger, multicenter cohorts with comprehensive clinical and functional analyses is warranted. Therefore, future studies incorporating larger cohorts and mechanistic analyses are warranted to validate these findings and clarify their clinical relevance.

Although the present study is exploratory and based on a limited number of cases, our findings are consistent with previous reports demonstrating that multiple immune checkpoint molecules, including PD-1, LAG-3, and TIM-3, can be co-expressed within the tumor microenvironment in gastric cancer. Such co-expression has been associated with complex immune regulatory states and may contribute to variability in response to immune checkpoint blockade therapy [19, 20]. These observations support the concept that immune checkpoint heterogeneity may represent an important biological feature, warranting further investigation in larger cohorts.

## Conclusion

The diversity of immune checkpoint molecules may reflect heterogeneity in immune regulatory states and could be associated with differential responses to ICI therapy. These findings are hypothesis-generating and may warrant further investigation into personalized ICI strategies based on immune checkpoint expression patterns.

## Data Availability

The datasets used and analysed during the current study are available from the corresponding author on reasonable request.
